# LncRNA ZFPM2‐AS1 promotes lung adenocarcinoma progression by interacting with UPF1 to destabilize ZFPM2

**DOI:** 10.1002/1878-0261.12631

**Published:** 2020-02-20

**Authors:** Shuhua Han, Dandan Cao, Jun Sha, Xiaoli Zhu, Dongqin Chen

**Affiliations:** ^1^ Department of Pulmonary Medicine Zhongda Hospital School of Medicine Southeast University Nanjing China; ^2^ The Affiliated Cancer Hospital of Nanjing Medical University & Jiangsu Cancer Hospital & Jiangsu Institute of Cancer Research China

**Keywords:** lung adenocarcinoma, mRNA decay, UPF1, ZFPM2, ZFPM2‐AS1

## Abstract

Lung adenocarcinoma (LUAD), a histological subclass of non‐small‐cell lung cancer, is globally the leading cause of cancer‐related deaths. Long noncoding RNAs (lncRNAs) are emerging as cancer regulators. Zinc finger protein multitype 2 antisense RNA 1 (ZFPM2‐AS1) is an oncogene in gastric cancer, but its functions have not been investigated in LUAD. We showed that ZFPM2‐AS1 expression is high in LUAD samples based on GEPIA database (http://gepia.cancer-pku.cn/) and validated ZFPM2‐AS1 upregulation in LUAD cell lines. Functionally, ZFPM2‐AS1 facilitated proliferation, invasion, and epithelial‐to‐mesenchymal transition of LUAD cells. Thereafter, we found that ZFPM2 was negatively regulated by ZFPM2‐AS1, and identified the suppressive effect of ZFPM2 regulation by ZFPM2‐AS1 on LUAD progression. Mechanistically, we showed that ZFPM2‐AS1 interacted with up‐frameshift 1 (UPF1) to regulate mRNA decay of ZFPM2. Rescue assays *in vitro* and *in vivo* confirmed that ZFPM2‐AS1 regulated LUAD progression and tumor growth through ZFPM2. Taken together, our findings demonstrate a role for the ZFPM2‐AS1–UPF1–ZFPM2 axis in LUAD progression, suggesting ZFPM2‐AS1 as a new potential target for LUAD treatment.

## Graphical abstract

The illustration of lncRNA ZFPM2‐AS1 promoted lung adenocarcinoma progression through cofunctioning with UPF1 to destabilize ZFPM2.

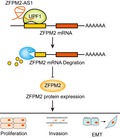

**Abbreviations**
CCK‐8: cell counting kit 8
DAPI: 4,6‐diamidino‐2‐phenylindole
EdU: 5‐Ethynyl‐2′‐deoxyuridine
EMT: epithelial‐to‐mesenchymal transition
FISH: fluorescence *in situ* hybridization
IF: immunofluorescence
LUAD: lung adenocarcinoma
MS: mass spectrometry
RIP: RNA immunoprecipitation
RT‐qPCR: quantitative real‐time PCR
shRNA: short hairpin RNA
siRNA: small interfering RNA
UPF1: up‐frameshift 1
ZFPM2: zinc finger protein multitype 2
ZFPM2‐AS1: zinc finger protein multitype 2 antisense RNA 1

## 1 Introduction

Lung adenocarcinoma (LUAD) is a major histological class of non‐small‐cell lung cancer (Herbst *et al.*, 2008), accounting for a large proportion of the global cancer‐related deaths (Siegel *et al.*, 2012). Statistics show that LUAD patients presented an average 5‐year survival rates < 20% (Lin *et al.*, 2016), despite that the advancing therapeutic tools improved the prognosis of certain subgroups of patients with LUAD (Kim *et al.*, 2015; Lin *et al.*, 2012). Therefore, deep‐going investigation on mechanism of LUAD progression is required to identify new biomarkers and improve the efficacy of molecular‐targeted therapies.

Long noncoding RNAs (lncRNAs), defined as non‐protein‐coding transcripts with more than 200 nucleotides, have been demonstrated to be novel participants in cancer‐related biological processes (Liu *et al.*, 2015; Prensner *et al.*, 2014; Wang *et al.*, 2016a; Wang *et al.*, 2016b). Also, increasing studies have revealed the regulatory functions of lncRNAs in LUAD (Lu *et al.*, 2017; Qiu *et al.*, 2015; Zhang *et al.*, 2017b). LncRNA zinc finger protein multitype 2 (ZFPM2) antisense RNA 1 (ZFPM2‐AS1) has been identified to exert promoting effect on gastric cancer progression through stabilizing MIF and inhibiting p53 pathway (Kong *et al.*, 2018), but whether it has an impact in LUAD remains unknown.

Mechanistically, mounting studies have illustrated that lncRNAs could regulate gene expressions at post‐transcriptional level through interacting with RNA binding proteins (RBPs) and regulating mRNA stability (Wang *et al.*, 2019a; Wen *et al.*, 2018). Interestingly, besides the effects of lncRNAs on stabilizing mRNAs, studies also showed that lncRNAs could regulate mRNA decay through interacting with certain RBPs in diseases and cancers. For example, LncRNA MEG3 induced mRNA decay of Shp through interacting with PTBP1 to facilitate cholestatic liver injury (Zhang *et al.*, 2017a). LINC01093 triggered GLI1 mRNA decay through the interaction with IGF2BP1 to suppress HCC progression (He *et al.*, 2019). Recent studies have reported that up‐frameshift 1 (UPF1), a crucial regulator in RNA degradation pathways (Azzalin and Lingner, 2006), can induce mRNA decay to suppress the expressions of genes encoding proteins opposing the undifferentiated and proliferative cell state (Lou *et al.*, 2014). Although studies have demonstrated the association of UPF1 with lncRNAs such as SNHG6 and MALAT1 in cancers (Li *et al.*, 2017; Wang *et al.*, 2019b), its correlation with ZFPM2‐AS1 has never been explored.

Zinc finger protein multitype 2, whose encoding protein is a transcriptional cofactor of GATA‐binding family, is known as an important regulator of the genes responsible for the development of diverse organs (Lu *et al.*, 1999). The involvement of ZFPM2 in regulating tumor pathogenesis has been demonstrated in several cancers, such as neuroblastoma and sex cord‐derived ovarian tumors (Hoene *et al.*, 2009; Laitinen *et al.*, 2000). Moreover, studies have suggested the tumor‐suppressive effect of ZFPM2 in cancers by demonstrating its effects on cell apoptosis and differentiation (Hyun *et al.*, 2009; Manuylov, Smagulova, and Tevosian, 2007). Nevertheless, never has the role of ZFPM2 been explored in LUAD.

Therefore, the present study aimed to explore the functional role and regulatory mechanism of ZFPM2‐AS1 in LUAD.

## 2 Materials and methods

### 2.1 Cell lines and cell culture

Four human LUAD cell lines (SPC‐A1, A549, H1299, and PC9) and the normal human bronchial epithelial cell line were provided by the Institute of Biochemistry and Cell Biology at the Chinese Academy of Sciences (Shanghai, China). The cells were cultured in RPMI‐1640 or DMEM (GIBCO‐BRL; Invitrogen, Carlsbad, CA, USA). 10% FBS (Gibco BRL, Grand Island, NY, USA) was used to supplement the medium with the treatment of 100 mg·mL^−1^ streptomycin and 100 U·mL^−1^ penicillin. The medium was kept in incubators at 37 °C with 5% CO_2_.

### 2.2 Cell transfection

Zinc finger protein multitype 2‐AS1, ZFPM2, and UPF1 were silenced by specific small interfering RNAs (siRNAs), termed si‐ZFPM2‐AS1#1/2/3, si‐ZFPM2#1/2/3, and si‐UPF1#1/2/3, with scramble siRNAs acting as negative control. For stable silence of ZFPM2‐AS1, short hairpin RNAs (shRNAs) targeting ZFPM2‐AS1 (sh‐ZFPM2‐AS1#1/2/3) and control shRNA (sh‐NC) were all synthesized by GenePharma (Shanghai, China). ZFPM2‐AS1 and UPF1 were overexpressed by pcDNA3.1 integrated with ZFPM2‐AS1 or UPF1 full sequences, termed pcDNA3.1/ZFPM2‐AS1 and pcDNA3.1/UPF1. The plasmids were purchased from GenePharma and were transfected as demanded into A549 or SPC‐A1 cells with the application of Lipofectamine 3000 (Invitrogen).

### 2.3 Quantitative real‐time PCR

Total RNAs isolated from LUAD cells were obtained with the application of TRIeasy™ Total RNA Extraction Reagent TRIeasy™ (YEASEN, Shanghai, China). The generation of cDNA was conducted by the use of Hifair™ II 1 st Strand cDNA Synthesis Kit (YEASEN). The evaluation of mRNA expression was conducted by using Hieff Unicon TaqMan multiplex qPCR master mix (YEASEN) with an ABI Prism 7500 Detection System (Applied Biosystems, Inc., Carlsbad, CA, USA). GAPDH acted as internal reference. Calculation of relative mRNA levels was conducted on the basis of 2^−△△ct^ method. Primers were as follows: ZFPM2‐AS1: 5′‐CAATGGGACTAAGCCAGGCA‐3′ (forward), 5′‐GGGCTCCACCAACAACCATA‐3′ (reverse); ZFPM2: (forward) 5′‐ACCGAAGGGATGTACCCTG‐3′, (reverse) 5′‐TCGTTGCCTCCCACTACAGT‐3′.

UPF1: 5′‐TCACGGCACAGCAGATCAACAAG‐3′ (forward), 5′‐CGGCTTCTCCAGGTCCTCCAG‐3′ (reverse); GAPDH: 5′‐GTCAACGGATTTGGTCTGTATT‐3′ (forward), 5′‐AGTCTTCTGGGTGGCAGTGAT‐3′ (reverse).

### 2.4 Cell proliferation assays

Lung adenocarcinoma cells were seeded into 96‐well plates and incubated for 0, 24, 48, 72, and 96 h, followed by the addition of cell counting kit 8 (CCK‐8; Dojindo Laboratories, Kumamoto, Japan). Optical density was determined with the use of a microplate reader (Bio‐Rad, Hercules, CA, USA).

5‐Ethynyl‐2′‐deoxyuridine (EdU) immunofluorescence (IF) staining was conducted with EdU kit (Roche, Mannheim, Germany). After the incubation with EdU for 5 h, cells were subjected to fixation with 4% paraformaldehyde, followed by the permeabilization in 0.5% Triton X‐100 diluted in PBS. Thereafter, LUAD cells were stained with Apollo staining solution and then incubated with 4,6‐diamidino‐2‐phenylindole (DAPI; Sigma‐Aldrich, St. Louis, MO, USA). Inverted fluorescence microscope (Carl Zeiss, Jena, Germany) was used to observe the EdU‐positive cells.

### 2.5 Cell invasion assay

To evaluate cell invasion, 24‐well transwell chamber (8 μm pore size; Corning Inc., Corning, NY, USA) coated with matrigel (BD biosciences, Sparks, MD, USA) was used. Transfected LUAD cells were seeded in the top insert in the nonserum medium, whereas the lower chamber was filled with the 10% FBS. After incubation for 1 day, a cotton swab was used to remove the cells that had not invaded through the pores, and the cells invading to the lower membrane surface were subjected to the staining in crystal violet and then photographed by a microscope. Invading cell number was calculated by using imagej software (National Institutes of Health, Bethesda, MD, USA).

### 2.6 Subcellular fractionation location

The nuclear and cytosolic fractions of RNAs were divided with the use of the PARIS Kit (Life Technologies, Carlsbad, CA, USA) following former description (Hu *et al.*, 2016). Quantitative real‐time PCR (RT‐qPCR) was used to determine the expression of ZFPM2‐AS1, with U6 and GAPDH used as internal references of cytoplasmic and nuclear RNA.

### 2.7 Luciferase reporter assay

The promoter of ZFPM2 was inserted into the pGL3 basic luciferase reporter vectors (Promega, Madison WI, USA). ZFPM2 promoter reporter was transfected with pcDNA3.1/ZFPM2‐AS1 or si‐ZFPM2‐AS1#1/2 (with pcDNA3.1 or si‐NC as negative controls) into 293T cells. The luciferase activity of ZFPM2 promoter reporter was normalized to Renilla luciferase reporter. One day after transfection, the detection of luciferase activity was conducted with the application of a Dual‐Luciferase Reporter Assay System (Promega).

### 2.8 Fluorescence *in situ* hybridization and immunofluorescence

After being washed and fixed in paraformaldehyde (Solarbio, Beijing, China), cells were subjected to the permeabilization in 0.5% Triton X‐100. For fluorescence *in situ* hybridization (FISH) assay, Cy3‐labeled probes of ZFPM2‐AS1 were used to detect the expression of ZFPM2‐AS1 in LAUD cells. Hybridization was conducted by utilizing fluorescent *in situ* Hybridization Kit (RIBO Bio, Guangzhou, China) in the dark environment for 12 h. For IF assay, LUAD cells were incubated with antibodies against UPF1, E‐cadherin, and N‐cadherin (all from Abcam, Cambridge, UK) for 2 h. The nuclei of LUAD cells were stained by DAPI. The observation was conducted by the confocal laser microscope (Olympus Optical, Tokyo, Japan).

### 2.9 Pull‐down assay and mass spectrometry analysis

Zinc finger protein multitype 2‐AS1 and the antisense control sequences were transcribed *in vitro* and subjected to biotin‐labeling applying T7 RNA polymerase (Roche Diagnostics, Indianapolis, IN, USA) and the Biotin RNA Labeling Mix (Roche). Then, the whole‐cell lysates from LUAD cells were subjected to the incubation for 1 h with the purified biotinylated transcripts. Thereafter, the complexes were isolated by the use of streptavidin agarose beads (Invitrogen). The retrieved proteins were examined by western blot analysis or subjected to the resolving in gradient gel electrophoresis, followed by the mass spectrometry (MS) analysis (TripleTOF 5600 LCMS; AB SCIEX, Carlsbad, CA, USA).

### 2.10 RNA immunoprecipitation

RNA immunoprecipitation (RIP) assays were carried out using the EZ‐Magna RIP RNA‐Binding Protein Immunoprecipitation Kit (Millipore, Billerica, MA, USA) according to the manufacturer’s instructions. For normal RIP, the antibody against UPF1 (Abcam) was used. The precipitated RNA was examined by RT‐qPCR and western blot analyses.

### 2.11 MS2‐based RNA immunoprecipitation

The MS2‐RIP was conducted referring to previous description (Gong and Maquat, 2011). The binding sites on the 3′UTR of ZFPM2 mRNA were obtained by comparing the binding motif of FUS from Starbase (http://starbase.sysu.edu.cn/) and the 3’UTR sequences of ZFPM2 mRNA. LUAD cells were transfected with pcDNA‐ZFPM2‐3′UTR‐MS2 (with the predicted binding sites) or pcDNA‐MS2. The immunoprecipitation was carried out utilizing anti‐GFP antibody or IgG and the Magna RIP RNA‐Binding Protein Immunoprecipitation Kit (Millipore) or Pierce™ Co‐Immunoprecipitation Kit (Thermo Fisher Scientific, Inc., Waltham, MA, USA). The precipitated RNA and protein levels were examined by RT‐qPCR and western blot.

### 2.12 Western blot

The protein extraction was obtained by the application of RIPA lysis buffer (Beyotime, Shanghai, China) with 1% phenylmethylsulfonyl fluoride (Roche, Basel, Switzerland). After the loading and separation of proteins by 10% SDS/PAGE, proteins were subjected to the shifting onto nitrocellulose membranes (Millipore). Following the sealing of membranes in block buffer BSA (5% w/v in PBS), membranes were incubated with primary antibodies for 12 h. Then, the signals were probes with the horseradish peroxidase‐conjugated secondary antibodies and the revealing was conducted by the ECL kit (Thermo Fisher Scientific, Inc.). GAPDH acted as internal control. Primary antibodies were as follows: anti‐E‐cadherin, anti‐N‐cadherin, anti‐UPF1, and anti‐GAPDH from Abcam; anti‐ZFPM2 from Biotechnology, Inc. (Santa Cruz, CA, USA).

### 2.13 *In vivo* xenograft tumor models

Animal experiments were conducted obeying the guidelines with the approval of the Institutional Animal Care and Use Ethics Committee of Zhongda Hospital, School of Medicine, Southeast University. The 6‐week‐old female BALB/c nude mice were injected with A549 cells transfected with pcDNA3.1, pcDNA3.1/ZFPM2‐AS1, or pcDNA3.1/ZFPM2‐AS1 + ZFPM2. The tumor growth was evaluated every 4 days. After 20 days, the mice were sacrificed for the resection of xenografts, and the tumor weight and volume were detected.

### 2.14 Statistical analysis

For data presentation, mean ± standard deviation (SD) was used. Data analyses were carried out by the application of spss 22.0 software (IBM, Armonk, NY, USA, SPSS, USA) and graphpad prism v5.01 (GraphPad, La Jolla, CA, USA) software. The differences of statistics between two groups or among over two groups were evaluated by two‐tailed Student's *t*‐tests or one‐way ANOVA. All experiments were conducted thrice. All assays were triplicated. A probability level < 0.05 was used to determine the statistical significance.

## 3 Results

### 3.1 ZFPM2‐AS1 was upregulated in LUAD, promoting proliferation in LUAD cells

**Figure 1:**
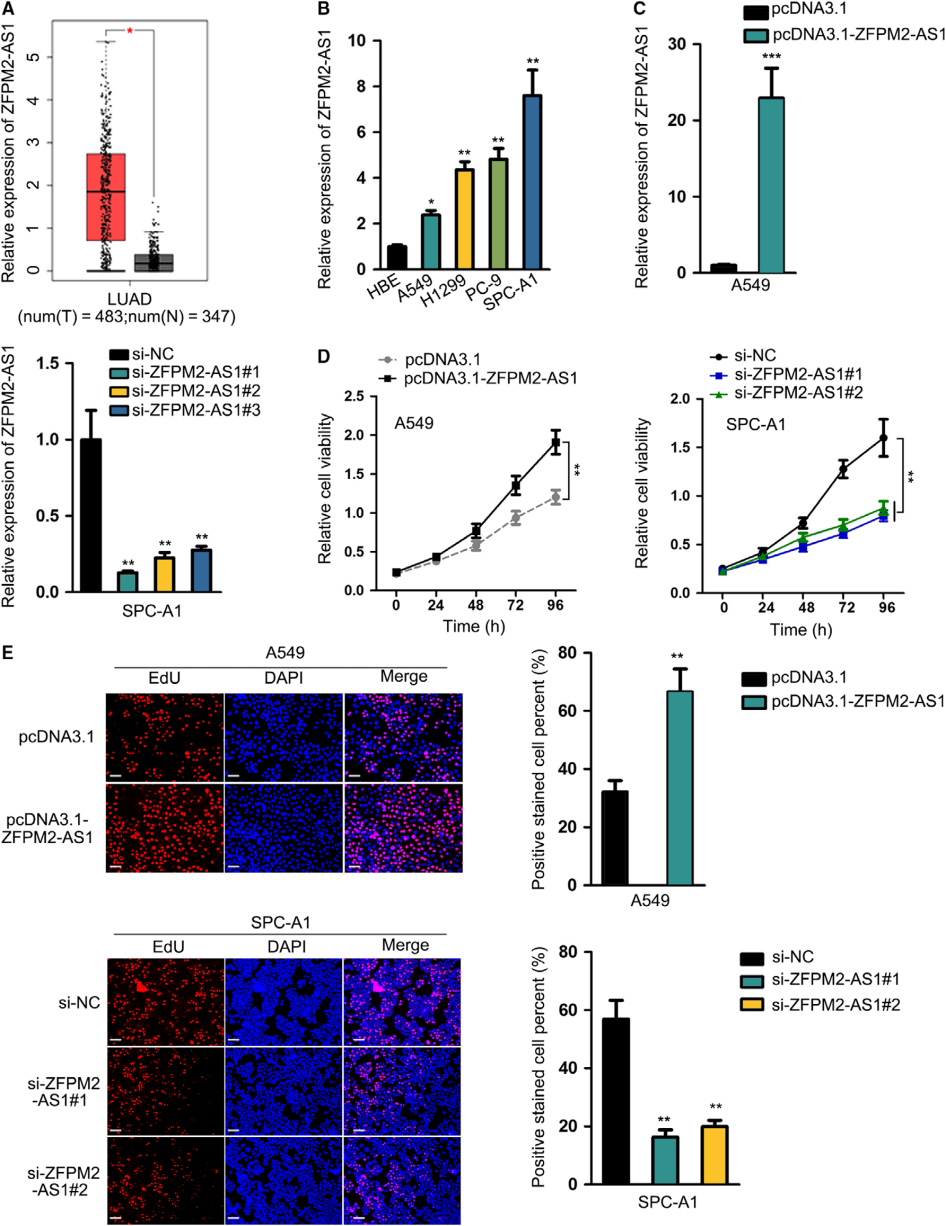
ZFPM2‐AS1 was upregulated in LUAD, promoting proliferation in LUAD cells. (A) Upregulation of ZFPM2‐AS1 in LUAD samples was obtained in GEPIA database. (B) RT‐qPCR results of the upregulation of ZFPM2‐AS1 in LUAD cell lines (mean ± SD; *n* = 6; one‐way ANOVA). (C) RT‐qPCR results of the overexpression of ZFPM2‐AS1 by pcDNA3.1/ZFPM2‐AS1 in A549 cells and the knockdown of ZFPM2‐AS1 by si‐ZFPM2‐AS1#1/2/3 in SPC‐A1 cells (mean ± SD; *n* = 6; Student’s *t*‐test and one‐way ANOVA). (D, E) CCK‐8 (mean ± SD; *n* = 6; two‐way ANOVA) and EdU assays (scale bar = 100 μm; mean ± SD; *n* = 6; Student’s *t*‐test and one‐way ANOVA) were used to assess proliferation of LUAD cells upon ZFPM2‐AS1 overexpression and knockdown. *P < 0.05, **P < 0.01, ***P < 0.001.

In order to figure out whether ZFPM2‐AS1 was associated with LUAD, we browsed GEPIA database. Data showed that ZFPM2‐AS1 exhibited high expression in 483 LUAD samples compared with 347 nontumor samples (Fig. 1A). Then, we tested ZFPM2‐AS1 expressions in cell lines. Results from RT‐qPCR displayed that ZFPM2‐AS1 expression was higher in LUAD cell lines than in normal cell line, and among LUAD cell lines, A549 presented the lowest ZFPM2‐AS1 expression while SPC‐A1 presented the highest (Fig. 1B). Thereafter, we designed *in vitro* gain‐of‐function and loss‐of‐function assays to investigate the impact of ZFPM2‐AS1 in LUAD. Based on the expression level of ZFPM2‐AS1 we identified in LUAD cells, we picked A549 cells to overexpress ZFPM2‐AS1 and selected SPC‐A1 cells to silence ZFPM2‐AS1. ZFPM2‐AS1 overexpression caused by pcDNA3.1/ZFPM2‐AS1 and ZFPM2‐AS1 knockdown by three siRNAs were validated by RT‐qPCR results, and si‐ZFPM2‐AS1#1 and si‐ZFPM2‐AS1#2 had better knockdown efficiency (Fig. 1C). We then observed that up‐regulated ZFPM2‐AS1 promoted cell proliferation and silenced ZFPM2‐AS1 inhibited cell proliferation (Fig. 1D,E). Jointly, results above implied that ZFPM2‐AS1 was upregulated in LUAD, promoting proliferation in LUAD cells.

### 3.2 ZFPM2‐AS1 promoted invasion and EMT in LUAD

**Figure 2:**
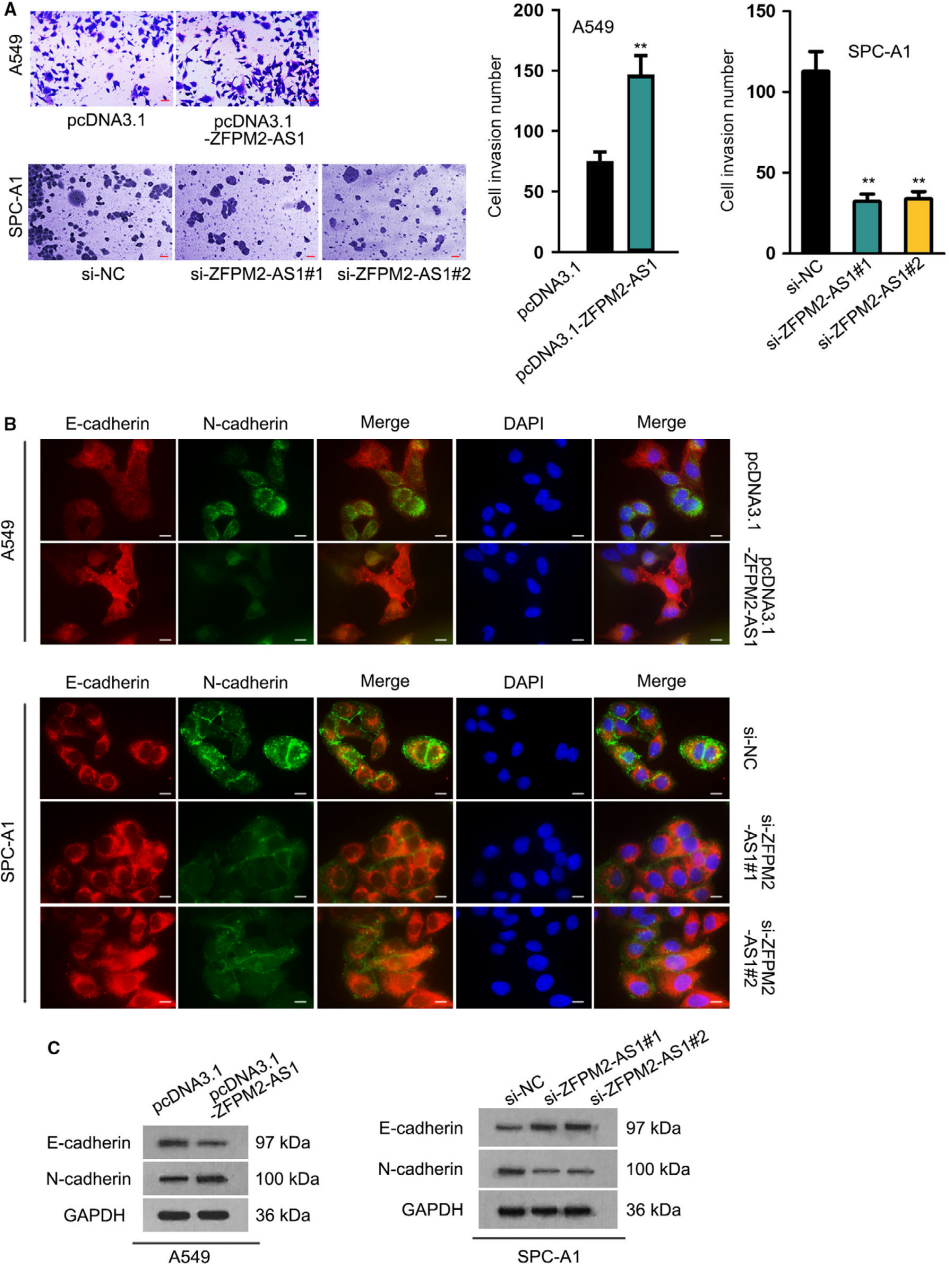
ZFPM2‐AS1 promoted invasion and EMT in LUAD. (A) Transwell invasion assay was used to assess LUAD cell invasive ability upon ZFPM2‐AS1 overexpression and knockdown (scale bar = 50 μm; mean ± SD; *n* = 6; Student’s *t*‐test and one‐way ANOVA). (B, C) IF (scale bar = 20 μm; *n* = 6) and western blot assays were used to evaluate the expressions of E‐cadherin and N‐cadherin upon ZFPM2‐AS1 overexpression and knockdown in LUAD cells. **P < 0.01.

Meanwhile, we detected the influence of ZFPM2‐AS1 on cell invasion in LUAD. Transwell invasion assay depicted that forced expression of ZFPM2‐AS1 induced invasion in A549 cells, whereas silenced expression of ZFPM2‐AS1 retarded invasion in SPC‐A1 cells (Fig. 2A). Additionally, epithelial‐to‐mesenchymal transition (EMT) progression was determined by examining the expressions of EMT markers upon ZFPM2‐AS1 up‐ or downregulation. IF staining assay demonstrated that overexpressing ZFPM2‐AS1 decreased E‐cadherin level and increased N‐cadherin level, while silencing ZFPM2‐AS1 had opposite effects (Fig. 2B). Same results were observed by western blot assay (Fig. 2C, Fig S2‐2C). Together, these data suggested that ZFPM2‐AS1 promoted invasion and EMT in LUAD.

### 3.3 ZFPM2, negatively regulated by ZFPM2‐AS1, was downregulated in LUAD and inhibited proliferation, invasion, and EMT

**Figure 3:**
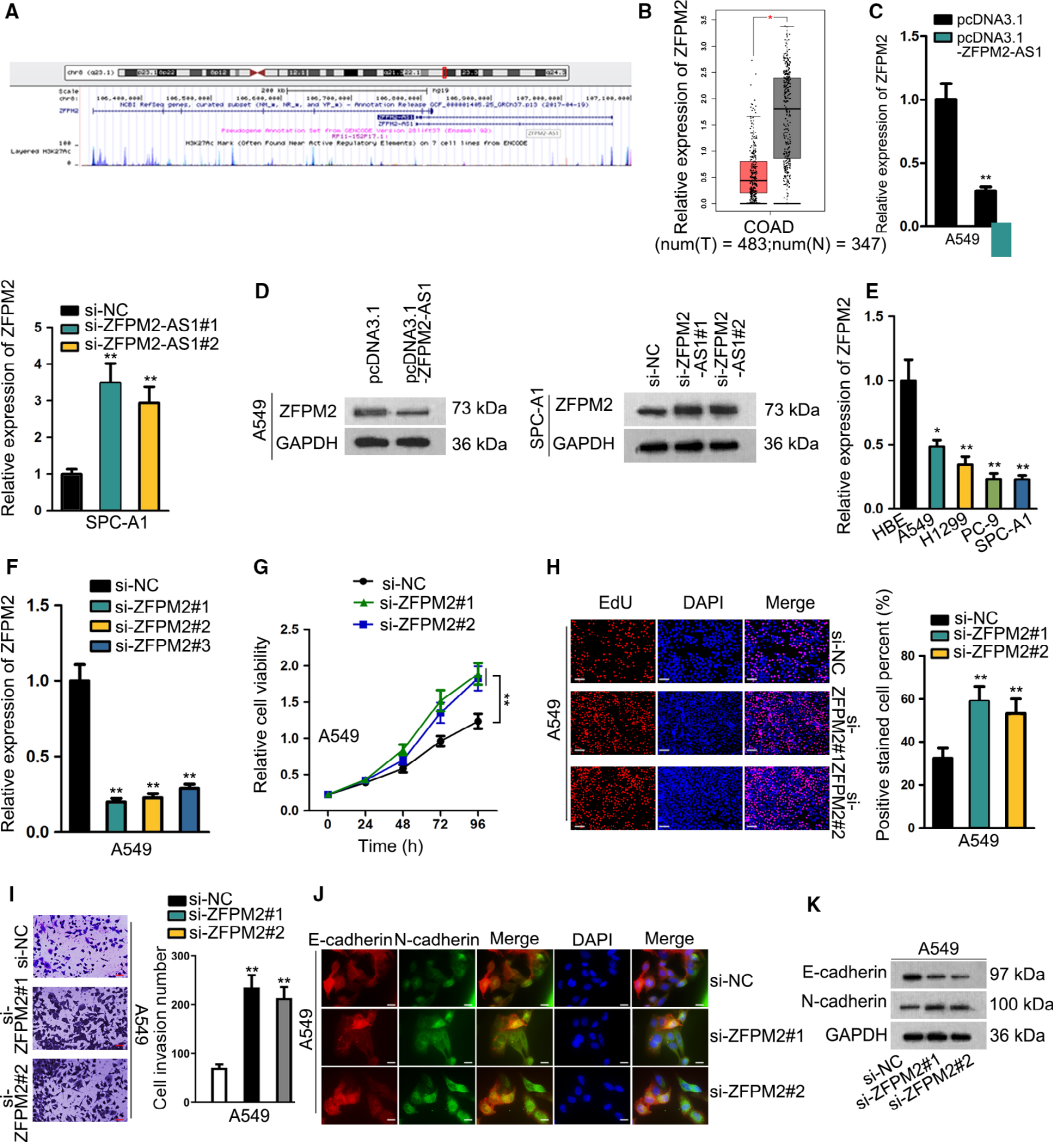
ZFPM2, negatively regulated by ZFPM2‐AS1, was downregulated in LUAD and inhibited proliferation, invasion, and EMT. (A) UCSC database showed that ZFPM2 was a nearby gene for ZFPM2‐AS1. (B) The downregulation of ZFPM2 in LUAD samples was obtained in TCGA database. (C, D) RT‐qPCR (mean ± SD; *n* = 6; Student’s *t*‐test and one‐way ANOVA) and western blot results of ZFPM2 expression upon ZFPM2‐AS1 overexpression and knockdown in LUAD cells. (E) Downregulation of ZFPM2 expression in LUAD cell lines was detected by RT‐qPCR results (mean ± SD; *n* = 6; one‐way ANOVA). (F) Knockdown of ZFPM2 by si‐ZFPM2#1/2/3 in A549 cells was confirmed by RT‐qPCR (mean ± SD; *n* = 6; one‐way ANOVA). (G–I) CCK‐8 (mean ± SD; *n* = 6; two‐way ANOVA), EdU (scale bar = 100 μm; mean ± SD; *n* = 6; one‐way ANOVA), and Transwell assays (scale bar = 50 μm; mean ± SD; *n* = 6; one‐way ANOVA) were used to evaluate the proliferative and invasive abilities of LUAD cells upon ZFPM2 silence. (J, K) IF staining (scale bar = 20 μm; *n* = 6) and western blot analyses were used to evaluate the expressions of E‐cadherin and N‐cadherin upon ZFPM2 knockdown in LUAD cells. *P < 0.05, **P < 0.01.

Then, we tried to explore the mechanism of ZFPM2‐AS1. LncRNAs often function in cancers through regulating target genes, and studies have shown that lncRNAs possess potential to regulate its nearby genes (Jiang *et al.*, 2019; Yan *et al.*, 2017). Through UCSC database (http://genome.ucsc.edu/), we identified ZFPM2 as a nearby gene for ZFPM2‐AS1 (Fig. 3A), indicating the regulatory potential of ZFPM2‐AS1 on ZFPM2. ZFPM2 has been suggested by previous studies to perform tumor‐suppressive roles in several cancers (Hyun *et al.*, 2009; Manuylov, Smagulova, and Tevosian, 2007), but never has it been investigated in LUAD. Therefore, we first examined the relation of ZFPM2 with LUAD. GEPIA data displayed a significant downregulation of ZFPM2 in LUAD samples in contrast to the normal samples (Fig. 3B). Next, we evaluated the influence of ZFPM2‐AS1 on ZFPM2 expression. Results of RT‐qPCR and western blot demonstrated that overexpression of ZFPM2‐AS1 reduced, whereas silence of ZFPM2‐AS1 induced the expression of ZFPM2 at mRNA and protein levels (Fig. 3C‐D and Fig S2‐3D). To further prove the effect of ZFPM2‐AS1 on ZFPM2 expression, we stably silenced ZFPM2‐AS1 in SPC‐A1 cell with specific shRNAs (Fig. S1A). And we found that protein level of ZFPM2 was enhanced a lot after stable silencing of ZFPM2‐AS1 (Figs. S1B, S2). In addition, we validated the overt downregulation of ZFPM2 in LUAD cell lines, among which A549 exhibited the highest expression of ZFPM2 (Fig. 3E).

Thereafter, we silenced endogenous expression of ZFPM2 in A549 cells to detect the functional role of ZFPM2 in LUAD. RT‐qPCR data confirmed the knockdown of ZFPM2 in A549 cells by three specific siRNAs, among which si‐ZFPM2#1 and si‐ZFPM2#2 presented better efficiency (Fig. 3F), so we chose these two siRNAs for subsequent loss‐of‐function assays. We observed that LUAD cell proliferation was facilitated in response to the silence of ZFPM2 (Fig. 3G,H). The invasive cells were increased in the presence of ZFPM2 silence (Fig. 3I). The expression of E‐cadherin was declined, and the expression of N‐cadherin was boosted upon the knockdown of ZFPM2 (Fig. 3J‐K, Fig. S2‐3K). In collection, results above indicated that ZFPM2, negatively regulated by ZFPM2‐AS1, was downregulated in LUAD and inhibited proliferation, invasion, and EMT.

### 3.4 ZFPM2‐AS1 potentially regulated ZFPM2 through interacting with UPF1

**Figure 4:**
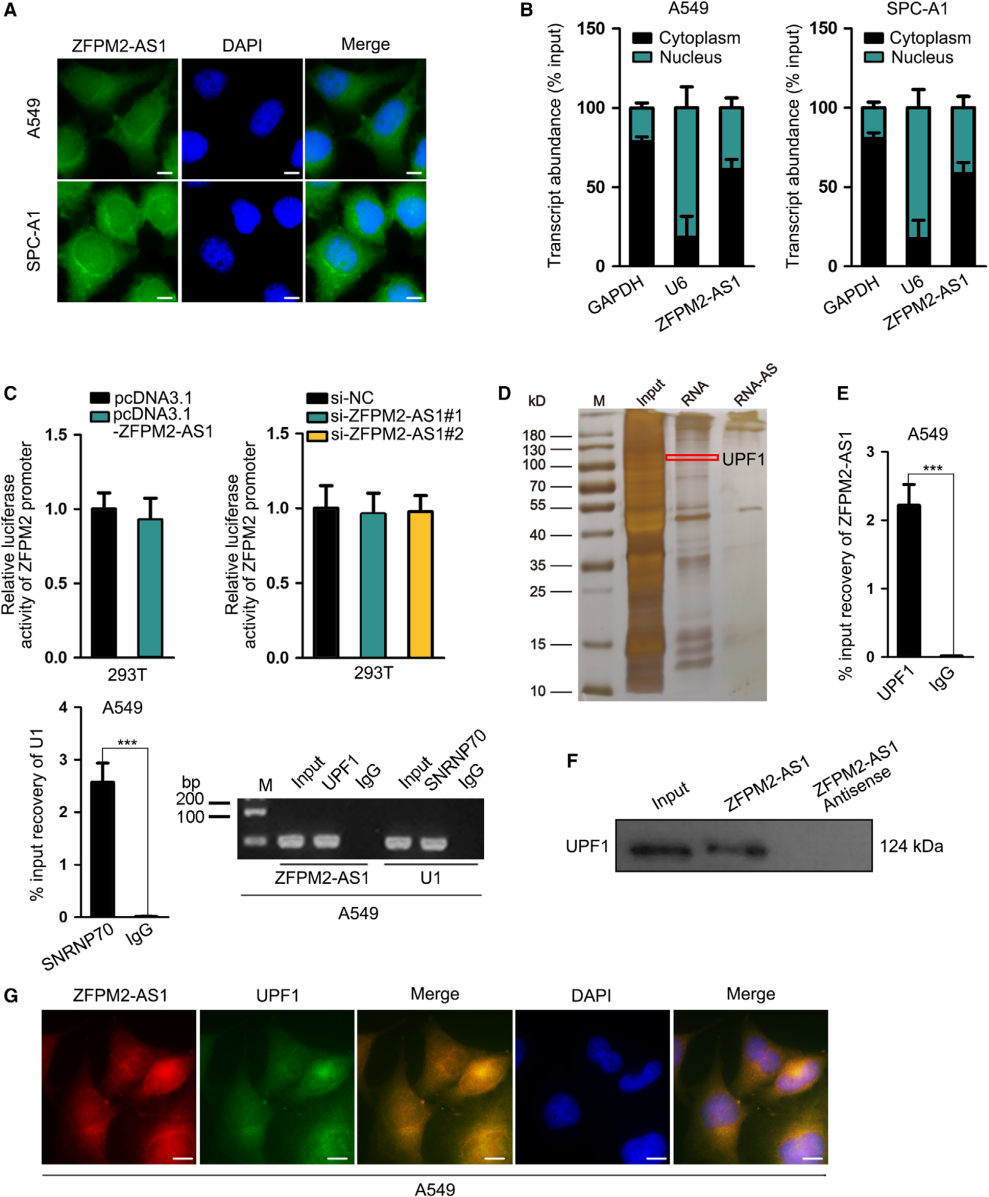
ZFPM2‐AS1 potentially regulated ZFPM2 through interacting with UPF1. (A, B) FISH (scale bar = 10 μm; *n* = 3) and subcellular fractionation assays were used to determine the cellular localization of ZFPM2‐AS1 in LUAD cells. (C) Luciferase reporter assay (mean ± SD; *n* = 6; Student’s *t*‐test and one‐way ANOVA) was used to assess the effect of ZFPM2‐AS1 on promoter transcription of ZFPM2. (D) Pull‐down assay followed by MS was used to identify the interacting partner for ZFPM2‐AS1. (E, F) RIP (mean ± SD; *n* = 6; Student’s *t*‐test) and western blot assay after pull‐down assays confirmed the interaction between ZFPM2‐AS1 and UPF1. (G) FISH assay (scale bar = 10 μm; *n* = 3) confirmed the overlapped expression of ZFPM2‐AS1 in cytoplasm of LUAD cells. ***P < 0.001, n.s: no significance.

In subsequence, we interrogated the mechanism behind ZPFM2‐AS1 negatively regulating ZFPM2 in LUAD. First, we identified the expression of ZFPM2‐AS1 mainly in cytoplasm of LUAD cells by FISH staining and subcellular fractionation (Fig. 4A,B). Further, FISH assay revealed that ZFPM2‐AS1 was downregulated in SPC‐A1 cells transfected with ZFPM2‐AS1‐specific siRNAs (Fig. S1C). It has been acknowledged that antisense transcripts, similar to characterized lncRNAs, exhibited *trans*‐ and *cis‐*regulatory functions on target genes (Huang *et al.*, 2016; Villegas and Zaphiropoulos, 2015), resulting in transcriptional activation or post‐transcriptional modulation on mRNA stability. The luciferase reporter assay demonstrated the noneffect of either ZFPM2‐AS1 overexpression or ZFPM2‐AS1 knockdown on the luciferase activity of ZFPM2 promoter reporter (Fig. 4C), indicating that ZFPM2‐AS1 might regulate ZFPM2 post‐transcriptionally. It has been proved that in cytoplasm, lncRNAs could interact with RBPs to regulate the expression of target genes (Wang *et al.*, 2019a; Wen *et al.*, 2018). Therefore, we analyzed the interacting proteins with ZFPM2‐AS1 by pull‐down assay followed by MS. Consequently, we found that UPF1 was one of the interacting partners for ZFPM2‐AS1 (Fig. 4D). Interestingly, UPF1 has been delineated to be a crucial regulator in RNA degradation pathways (Azzalin and Lingner, 2006), responsible for mRNA decay of the genes encoding proteins opposing the undifferentiated and proliferative cell state (Lou *et al.*, 2014). Therefore, we selected UPF1 for further investigation. RIP assay presented the abundance of ZFPM2‐AS1 in the precipitates of UPF1 antibody (Fig. 4E), and western blot following pull‐down assay depicted the enrichment of UPF1 protein in the products pulled down by ZFPM2‐AS1 rather than antisense ZFPM2‐AS1 (Fig. 4F), confirming the interaction of ZFPM2‐AS1 with UPF1. Also, the overlapped localization of ZFPM2‐AS1 and UPF1 protein expressions in cytoplasm further validated their interaction (Fig. 4G). Together, these results indicated that ZFPM2‐AS1 potentially regulated ZFPM2 through interacting with UPF1.

### 3.5 UPF1 was upregulated in LUAD and cofunctioned with ZFPM2‐AS1 to interact with ZFPM2

**Figure 5:**
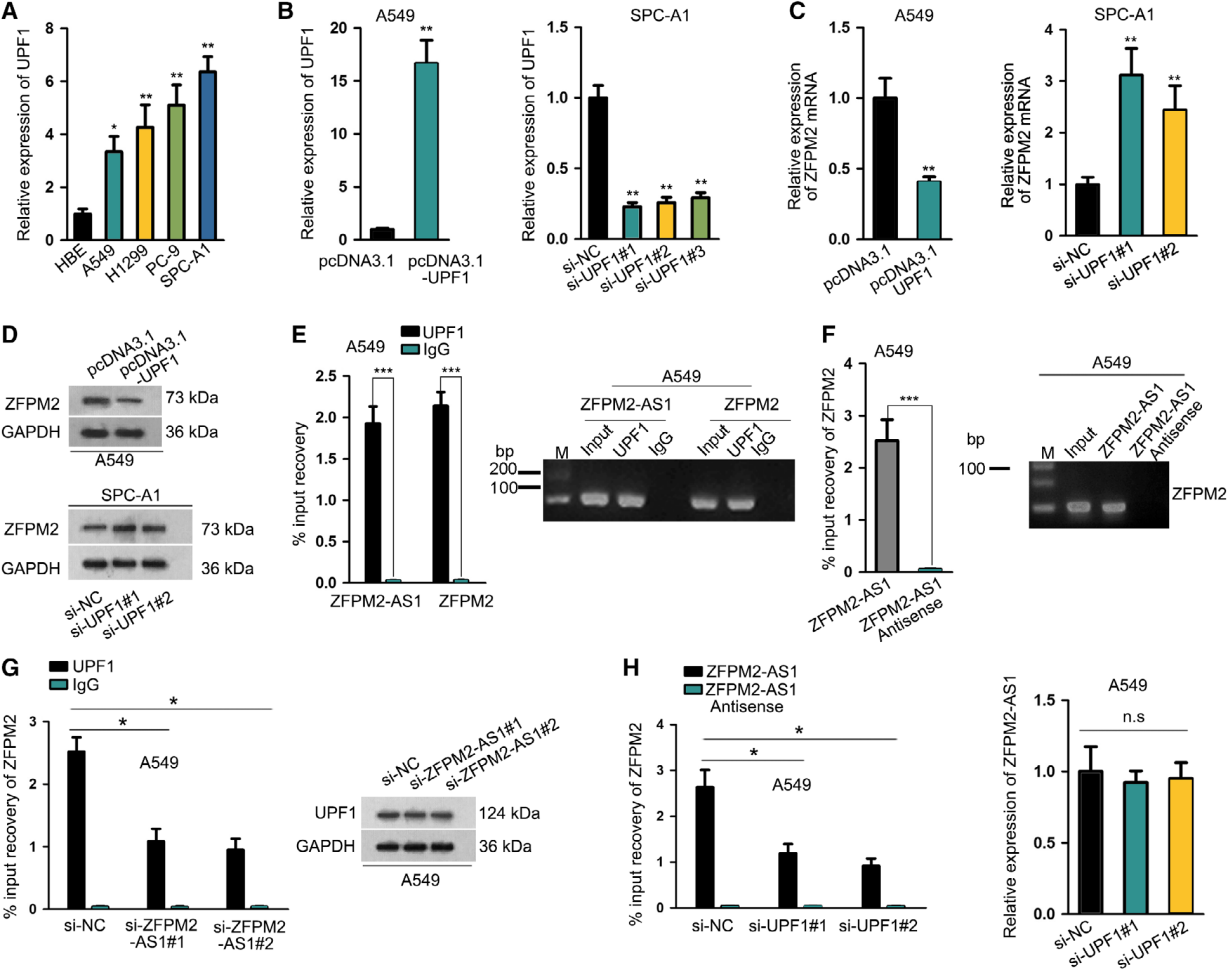
UPF1 was upregulated in LUAD and cofunctioned with ZFPM2‐AS1 to interact with ZFPM2. (A) RT‐qPCR results confirmed the upregulation of UPF1 in LUAD cell lines (mean ± SD; *n* = 6; one‐way ANOVA). (B) The overexpression and knockdown of UPF1 were confirmed by RT‐qPCR analysis (mean ± SD; *n* = 6; Student’s *t*‐test and one‐way ANOVA). (C, D) The effect of UPF1 on ZFPM2 expression was determined by RT‐qPCR (mean ± SD; *n* = 6; Student’s *t*‐test and one‐way ANOVA) and western blot analyses. (E, F) RIP (mean ± SD; *n* = 6; Student’s *t*‐test) and pull‐down assays (mean ± SD; *n* = 6; Student’s *t*‐test and one‐way ANOVA) were used to evaluate the interaction between UPF1 and ZFPM2 mRNA. (G, H) RIP assay (mean ± SD; *n* = 6; Student’s *t*‐test and one‐way ANOVA) and pull‐down assay (mean ± SD; *n* = 6; Student’s *t*‐test and one‐way ANOVA) showed that silencing ZFPM2‐AS1 interfered with the interaction of UPF1 with ZFPM2 mRNA and vice versa. Western blot and RT‐qPCR analyses confirmed that UPF1 had no effect on ZFPM2‐AS1 expression and vice versa. *P < 0.05, **P < 0.01, ***P < 0.001, n.s: no significance.

Then, we examined whether UPF1 had an impact on ZFPM2 expression. Before that, we confirmed the upregulation of UPF1 in LUAD cell lines (Fig. 5A). We overexpressed UPF1 in A549 cells and knocked it down in SPC‐A1 cells, which was confirmed by the results of RT‐qPCR (Fig. 5B). We observed the decrease of ZFPM2 mRNA and protein expression by UPF1 overexpression and the opposite results by UPF1 silence through RT‐qPCR and western blot (Fig. 5C‐D, Fig. S2‐5D). Furtherly, we investigated whether UPF1 participated in the interaction between ZFPM2‐AS1 and ZFPM2. RIP assay demonstrated that both ZFPM2‐AS1 and ZFPM2 mRNA could be immunoprecipitated by anti‐UPF1 (Fig. 5E). Pull‐down assay validated that ZFPM2‐AS1, rather than antisense ZFPM2‐AS1, could pull down ZFPM2 mRNA (Fig. 5F). These results implied that ZFPM2‐AS1, ZFPM2, and UPF1 formed a binding complex in LUAD cells. Additionally, we found that silencing ZFPM2‐AS1 impaired the interaction between UPF1 and ZFPM2 without reducing the expression of UPF1 (Fig. 5G, Fig. S2‐5G). Likewise, silencing UPF1 impaired the interaction between ZFPM2‐AS1 and ZFPM2 without reducing the expression of ZFPM2‐AS1 (Fig. 5H). These data suggested that ZFPM2‐AS1 and UPF1 functioned synergistically to interact with ZFPM2.

### 3.6 ZFPM2‐AS1 cofunctioned with UPF1 to destabilize ZFPM2 mRNA

**Figure 6:**
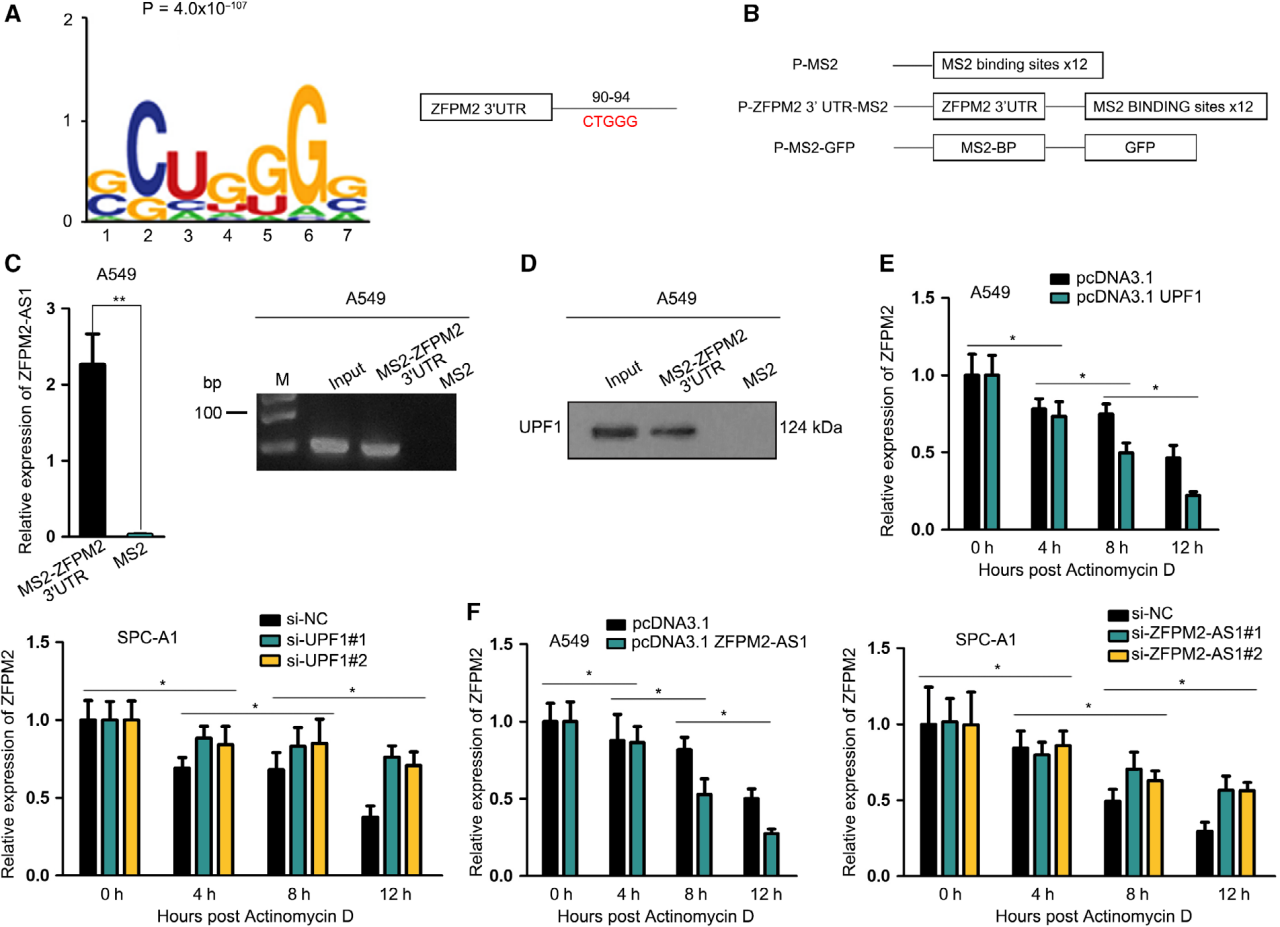
ZFPM2‐AS1 cofunctioned with UPF1 to destabilize ZFPM2 mRNA. (A) The binding motif of UPF1 and potential binding sites for UPF1 on ZFPM2 mRNA at 3′UTR region were obtained from Starbase. (B) The construction of MS2 vector containing ZFPM2 3′UTR region (mean ± SD; *n* = 6; Student’s *t*‐test). (C‐D) MS2‐RIP and MS2‐CoIP assays confirmed the interaction of UPF1 and ZFPM2‐AS1 with ZFPM2 mRNA at the binding sites in 3′UTR region. (E, F) LUAD cells were treated with actinomycin D to block the mRNA generation. RT‐qPCR analysis was used to evaluate the mRNA stability of ZFPM2 upon the overexpression and knockdown of UPF1 and ZFPM2‐AS1 (mean ± SD; *n* = 6; Student’s *t*‐test and one‐way ANOVA). *P < 0.05, **P < 0.01.

Furthermore, we explored the detailed mechanism whereby ZFPM2‐AS1/UPF2 regulated ZFPM2. As mentioned above, previous studies have demonstrated that UPF1 was a key regulator of mRNA decay for certain tumor‐suppressive genes (Lou *et al.*, 2014). Based on this, we hypothesized that ZFPM2‐AS1 cofunctioned with UPF1 to regulate the mRNA decay of ZFPM2. To validate our speculation, we first detected the detailed interaction of UPF1 and ZFPM2‐AS1 with ZFPM2 mRNA. Through Starbase, we identified the potential binding sites on the 3′ untranslated region (3′UTR) for UPF1 by comparing the binding motif of UPF1 and 3′UTR sequences of ZFPM2 (Fig. 6A). Then, we carried out MS2‐RIP by inserting the 3′UTR region containing the predicted binding sequences into MS2 plasmids (Fig. 6B). Results of RT‐qPCR and western blot followed by MS2‐RIP and MS2‐CoIP assays confirmed that ZFPM2‐AS1 and UPF1 protein were both enriched in MS2‐ZFPM2 3'UTR group compared with MS2 control group (Fig. 6C‐D, Fig. S2‐6D), confirming the interaction of UPF1 and ZFPM2‐AS1 with ZFPM2 mRNA at the predicted sites in 3′UTR region. Moreover, actinomycin D was added to block mRNA generation and ZFPM2 mRNA level was detected by RT‐qPCR every 4 hours. As a result, we found that overexpressing UPF1 or ZFPM2‐AS1 shortened the half‐life of ZFPM2 mRNA, whereas silencing UPF1 or ZFPM2‐AS2 exhibited opposite impact (Fig. 6E,F). In collection, these results suggested that ZFPM2‐AS1 cofunctioned with UPF1 to destabilize ZFPM2 mRNA.

### 3.7 ZFPM2‐AS1 regulated LUAD progression *in vitro* and *in vivo* through UPF1/ZFPM2 axis

**Figure 7:**
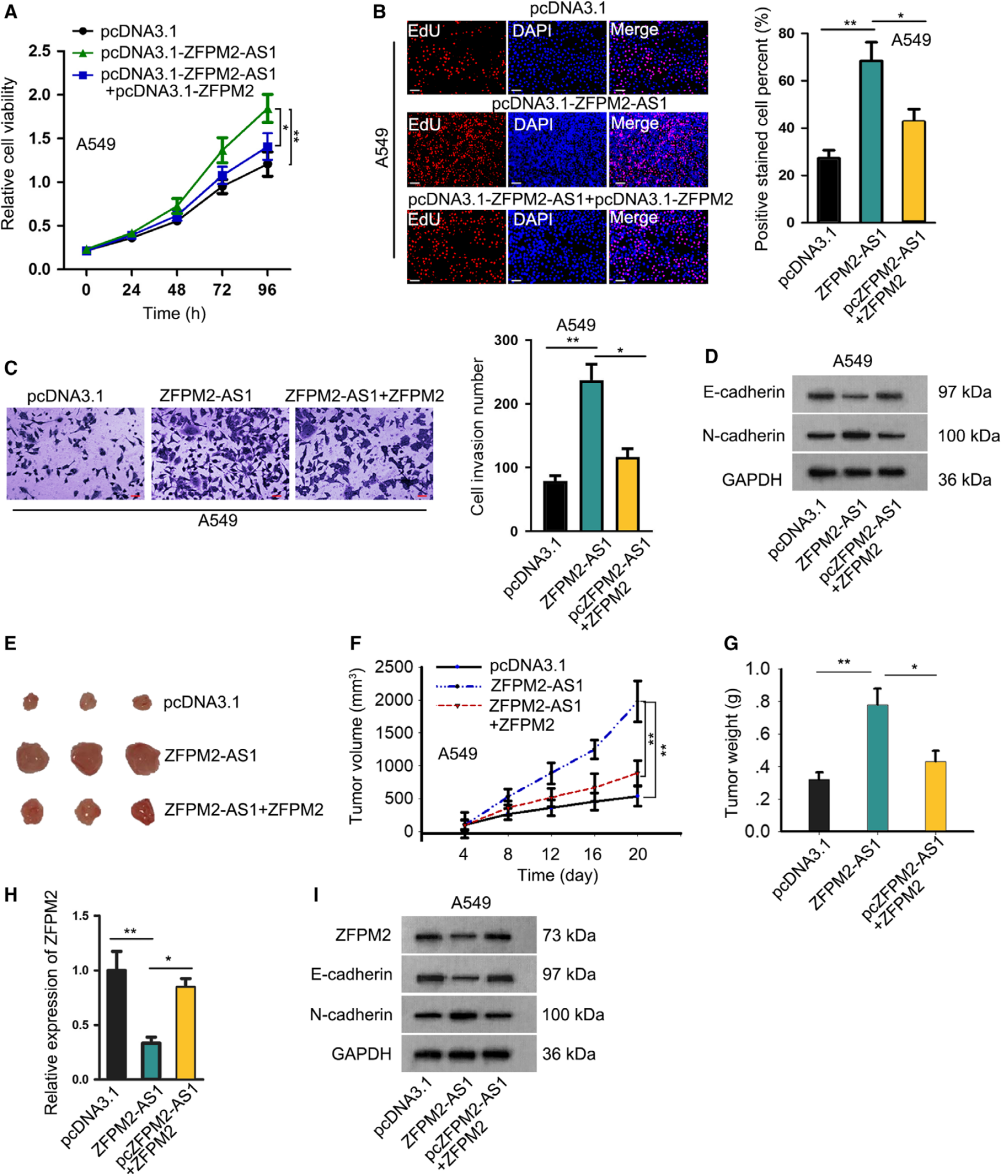
ZFPM2‐AS1 regulated LUAD progression *in vitro* and *in vivo* through UPF1/ZFPM2 axis. A549 cells were transfected with pcDNA3.1, pcDNA3.1/ZFPM2‐AS1, or pcDNA3.1/ZFPM2‐AS1 + ZFPM2, for *in vitro* and *in vivo* assays. (A–C) CCK‐8 (mean ± SD; *n* = 6; two‐way ANOVA), EdU (scale bar = 100 μm; mean ± SD; *n* = 6; one‐way ANOVA), and Transwell invasion assays (scale bar = 50 μm; mean ± SD; *n* = 6; one‐way ANOVA) were used to assess cell proliferation and invasion of A549 cells in each group. (D) Western blot analysis was used to evaluate the expressions of E‐cadherin and N‐cadherin in each group. (E) A549 cells with different transfection were injected into nude mice, and the growth of xenografts was evaluated over time. Pictures of xenografts from each group were taken. (F, G) The growth curve (mean ± SD; *n* = 6; two‐way ANOVA) and final tumor weight of the xenografts (mean ± SD; *n* = 6; one‐way ANOVA) in each group. (H) ZFPM2 expression from the xenografts in each group was evaluated by RT‐qPCR analysis (mean ± SD; *n* = 6; one‐way ANOVA). (I) Western blot analysis was used to evaluate the expression of ZFPM2, E‐cadherin, and N‐cadherin from the tumors of mice in each group. *P < 0.05, **P < 0.01.

Finally, we tried to investigate whether ZFPM2‐AS1 regulated LUAD progression through ZFPM2 by unfolding the rescue assays *in vitro* and *in vivo*. Consequently, the facilitative effect of ZFPM2‐AS1 overexpression on cell proliferation and invasion was abrogated by ZFPM2 overexpression (Fig. 7A–C). In addition, overexpressing ZFPM2 could reverse the decrease of E‐cadherin and increase of N‐cadherin caused by ZFPM2‐AS1 overexpression in A549 cells (Fig. 7D, Fig. S2‐7D). Thereafter, we grouped nude mice in three with the respective injection of A549 cells transfected with pcDNA3.1, pcDNA3.1‐ZFPM2‐AS1, or pcDNA3.1‐ZFPM2‐AS1 + ZFPM2. The growth of xenografts in mice was detected every 4 days. As a result, we validated that overexpression of ZFPM2‐AS1 promoted tumor growth *in vivo*, and forced expression of ZFPM2 could abrogate the facilitative effect of ZFPM2‐AS1 overexpression (Fig. 7E–G). Also, the expression of ZFPM2 mRNA and protein level in tumors was reduced by ZFPM2‐AS1 overexpression, which could be recovered by the cotransfection of pcDNA3.1‐ZFPM2 (Fig. 7H‐I, Fig. S2‐7I). Same results were observed in the protein level of E‐cadherin, whereas opposite results were observed in the protein level of N‐cadherin (Fig. 7I). In conclusion, these data suggested that ZFPM2‐AS1 regulated LUAD progression *in vitro* and *in vivo* through ZFPM2.

## 4 Discussion

Due to the unsatisfactory survival rate and high mortality of LUAD (Lin *et al.*, 2016; Siegel *et al.*, 2012), further improvement of therapy for LUAD is necessitated. LncRNAs have been identified as crucial participators in cancer‐related biological processes (Liu *et al.*, 2015; Prensner *et al.*, 2014; Wang *et al.*, 2016a; Wang *et al.*, 2016b), including in LUAD (Lu *et al.*, 2017; Qiu *et al.*, 2015; Zhang *et al.*, 2017b), indicating lncRNAs as novel promising molecular targets in LUAD. We were interested in the role of lncRNA ZFPM2‐AS1 because it was demonstrated by a previous report to be an carcinogen in gastric cancer (Kong *et al.*, 2018), but its role remains unexplored in LUAD. Accordingly, we discovered the elevation of ZFPM2‐AS1 expression in LUAD samples from TCGA database and confirmed its upregulation in LUAD cell lines. Functional assays indicated that ZFPM2‐AS1 exerted promoting function on proliferation, invasion, and EMT in LUAD cells.

Based on the known knowledge that lncRNAs can regulate target gene expression to realize its function in cancers and that lncRNAs could potentially regulate its nearby genes (Jiang *et al.*, 2019; Yan *et al.*, 2017), we browsed UCSC database and found that ZFPM2 was a nearby gene for ZFPM2‐AS1, suggesting that ZFPM2‐AS1 might regulate ZFPM2 expression. Formerly, studies have shown that ZFPM2 was involved in tumor pathogenesis in several cancers (Hoene *et al.*, 2009; Laitinen *et al.*, 2000), and have suggested the tumor‐suppressive effect of ZFPM2 (Hyun *et al.*, 2009; Manuylov, Smagulova, and Tevosian, 2007). Therefore, we focused on the exploration of ZFPM2. We firstly discovered through TCGA database that ZFPM2 was pronouncedly downregulated in LUAD samples, and validated that ZFPM2‐AS1 negatively regulated ZFPM2 expression. Also, we confirmed the low expression of ZFPM2 in LUAD cells and suggested that knockdown of ZFPM2 facilitated LUAD progression *in vitro*, firstly suggesting the tumor‐suppressive role of ZFPM2 in LUAD.

Furthermore, we identified the cytoplasmic expression of ZFPM2‐AS1 in LUAD cells and the noneffect of ZFPM2‐AS1 on ZFPM2 promoter transcription, suggesting that ZFPM2‐AS1 regulated ZFPM2 at post‐transcriptional level. Previous studies have illustrated the post‐transcriptional regulation of lncRNAs on gene expressions through interacting with RNA binding proteins and regulating mRNA stability (Wang *et al.*, 2019a; Wen *et al.*, 2018). Herein, we found through pull‐down and MS analysis that UPF1 was an interacting partner with ZFPM2‐AS1. We selected UPF1 because former studies have revealed that UPF1 could regulate mRNA decay of the genes exerting opposing effects on the undifferentiated and proliferative cell state (Lou *et al.*, 2014). In our study, we first confirmed the upregulation of UPF1 in LUAD cells and negative regulation of UPF1 on ZFPM2 expression. Then, we validated the interaction of ZFPM2 with UPF1 and ZFPM2‐AS1. Moreover, we found that inhibiting ZFPM2‐AS1 had no effect on UPF1 expression but could interfere with the interaction of UPF1 with ZFPM2 and vice versa, indicating that ZFPM2‐AS1 and UPF1 functioned synergistically regulated ZFPM2 expression. Previous studies have showed that lncRNAs could regulate mRNA decay through interacting with certain RBPs in diseases and cancers (He *et al.*, 2019; Zhang *et al.*, 2017a). According to these previous findings, our study firstly proved that ZFPM2‐AS1 interacted with UPF1 to regulate ZFPM2 mRNA decay. Finally, *in vitro* and *in vivo* rescue assays confirmed that ZFPM2‐AS1 regulated LUAD progression through ZFPM2.

In conclusion, our study firstly identified the oncogenic role of ZFPM1‐AS1 in LUAD and demonstrated a new mechanism that ZFPM2‐AS1 cofunctioned with UPF1 to destabilize ZFPM2 mRNA, indicating ZFPM2‐AS1 as a novel promising therapeutic target in LUAD.

## 5 Conclusion

Zinc finger protein multitype 2‐AS1 promoted LUAD cell growth, migration, and EMT process, thus exerting oncogenic functions. Mechanistically, ZFPM2‐AS1 interacted with UPF1 to promote ZFPM2 mRNA decay. These findings suggested the potential research value of ZFPM2‐AS1 as a promising therapeutic target in LUAD.

## Conflict of interest

The authors declare no conflict of interest.

## Author contributions

SH designed this study and analyzed the data. DC, JS, XZ, and DC were responsible for experiment record, figures and original article writing. All authors contributed to manuscript review and approved the final manuscript.

## Supporting information


**Fig. S1.** The expression of ZFPM2‐AS1 and ZFPM2 was examined after silencing of ZFPM2‐AS1. (A) Stable silence of ZFPM2‐AS1 in SPC‐A1 cell with specific shRNAs (mean ± SD; *n* = 6; one‐way ANOVA). (B) Protein level of ZFPM2 was assessed in SPC‐A1 cells after stable silencing of ZFPM2‐AS1. (C) FISH assay of ZFPM2‐AS1 in SPC‐A1 cells transfected with ZFPM2‐AS1‐specific siRNAs (scale bar = 20 μm; *n* = 3). **P < 0.01.

**Fig. S2.** Original protein bands.

## Data Availability

Research data are available.
