# Supplementary figures and images for: LncRNA ZFPM2‐AS1 promotes lung adenocarcinoma progression by interacting with UPF1 to destabilize ZFPM2
Source: Mol Oncol. 2020 Feb 20;14(5):1074–88. doi: 10.1002/1878-0261.12631 (PMC7191191; doi:10.1002/1878-0261.12631)

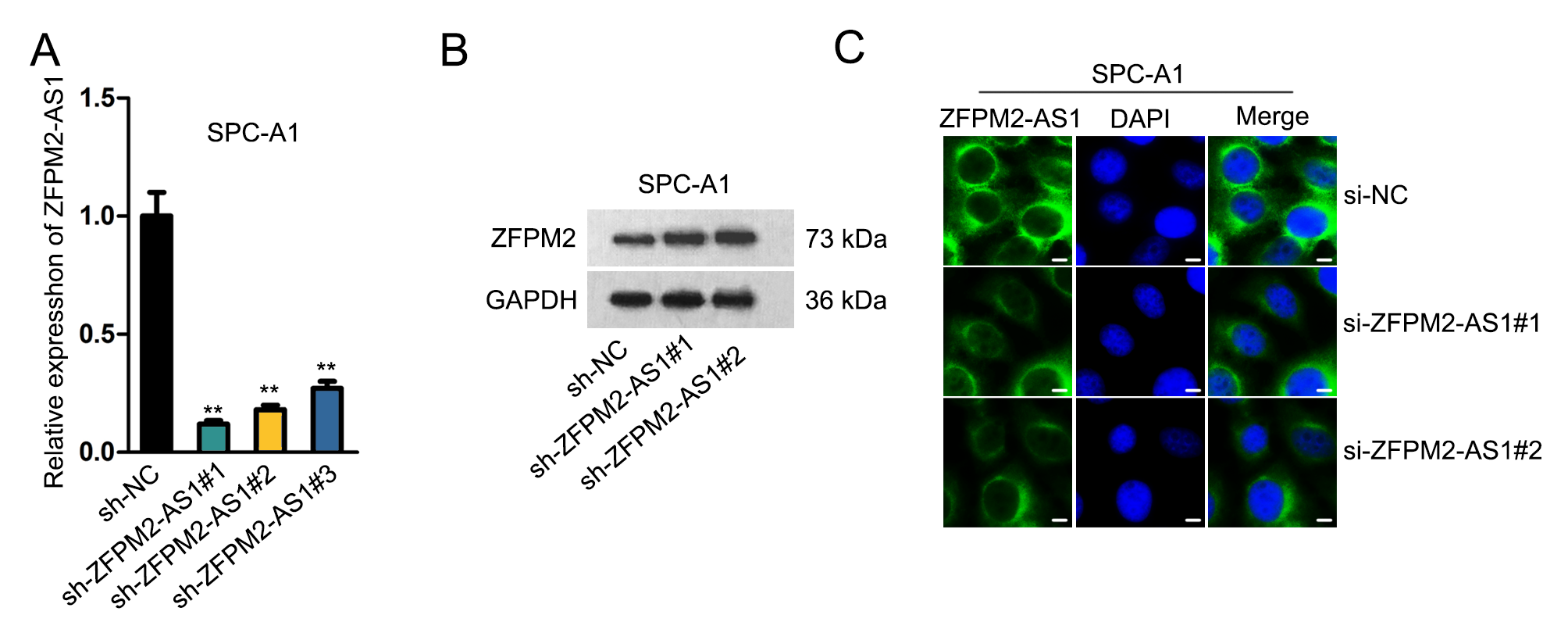

Supplement: Supplementary file 1 — Fig. S1. The expression of ZFPM2‐AS1 and ZFPM2 was examined after silencing of ZFPM2‐AS1. (A) Stable silence of ZFPM2‐AS1 in SPC‐A1 cell with specific shRNAs (mean ± SD; n = 6; one‐way ANOVA). (B) Protein level of ZFPM2 was assessed in SPC‐A1 cells after stable silencing of ZFPM2‐AS1. (C) FISH assay of ZFPM2‐AS1 in SPC‐A1 cells transfected with ZFPM2‐AS1‐specific siRNAs (scale bar = 20 μm; n = 3). **P < 0.01. [file MOL2-14-1074-s001.tif]

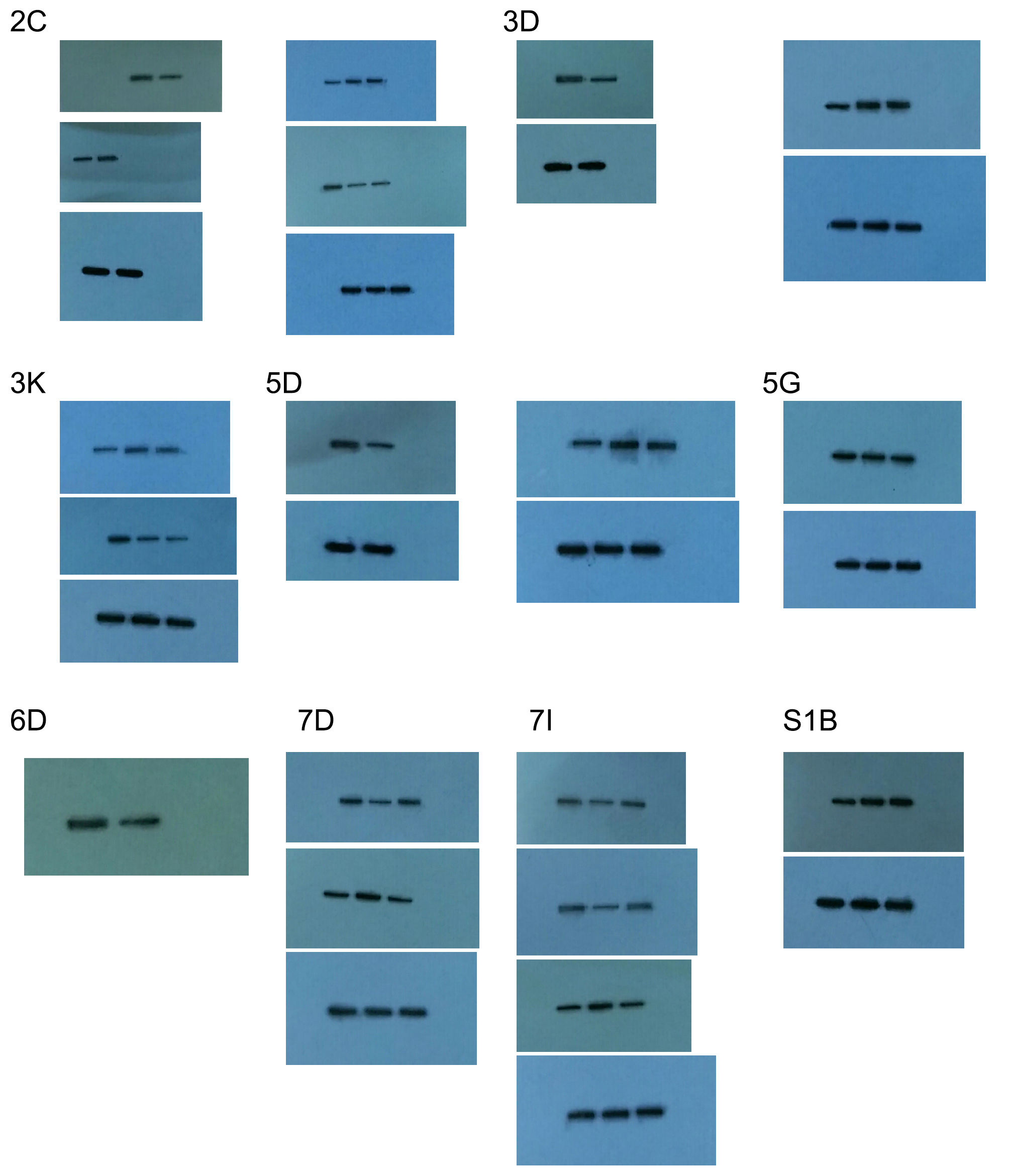

Supplement: Supplementary file 2 — Fig. S2. Original protein bands. [file MOL2-14-1074-s002.tif]
